# Arbitrarily rotating polarization direction and manipulating phases in linear and nonlinear ways using programmable metasurface

**DOI:** 10.1038/s41377-024-01513-2

**Published:** 2024-07-18

**Authors:** Wei Liu, Si Ran Wang, Jun Yan Dai, Lei Zhang, Qiao Chen, Qiang Cheng, Tie Jun Cui

**Affiliations:** 1 State Key Laboratory of Millimeter Waves, Southeast University, Nanjing, 210096 China; 2 Institute of Electromagnetic Space, Southeast University, Nanjing, 210096 China; 3 Frontiers Science Center for Mobile Information Communication and Security, Southeast University, Nanjing, 210096 China; 4 Electrical Engineering Department, Chalmers University of Technology, Gothenburg, 41258 Sweden

**Keywords:** Photonic devices, Frequency combs, Metamaterials

## Abstract

Independent controls of various properties of electromagnetic (EM) waves are crucially required in a wide range of applications. Programmable metasurface is a promising candidate to provide an advanced platform for manipulating EM waves. Here, we propose an approach that can arbitrarily control the polarization direction and phases of reflected waves in linear and nonlinear ways using a stacked programmable metasurface. Further, we extend the space-time-coding theory to incorporate the dimension of polarization, which provides an extra degree of freedom for manipulating EM waves. As proof-of-principle application examples, we consider polarization rotation, phase manipulation, and beam steering at linear and nonlinear frequencies. For validation, we design, fabricate, and measure a metasurface sample. The experimental results show good agreement with theoretical predictions and simulations. The proposed approach has a wide range of applications in various areas, such as imaging, data storage, and wireless communication.

## Abstract (web summary)

The polarization direction, beam steering, and frequency of the reflected wave are controlled by the STPC matrix.

## Graphical abstract

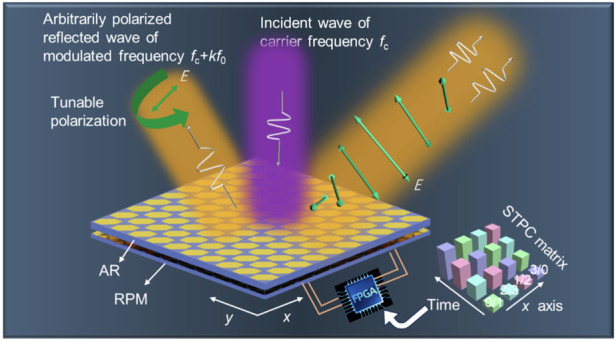

## Introduction

Recently, metasurfaces composed of engineered two-dimensional (2D) micro-structures have emerged as a promising platform for the manipulation of electromagnetic (EM) waves^1,2^ due to their inherent advantages such as low profile, low loss, and potential malleability. Consequently, they have attracted significant attention from researchers, leading to applications including cloaking^3^, abnormal refraction^4^, and absorption^5^. In 2014, Cui et al. introduced the concept of digital coding and programmable metasurfaces^6^, which allows for the manipulation of complex waves by modifying the discretized encoding sequence. This approach simplifies the design and optimization process of metasurfaces, and its application has been extensively explored in various fields^7–14^, such as diffuse scattering^7^, imager^10^, and reconfigurable intelligent surface^13,14^. It is important to note that the above metasurfaces only utilize spatial phase encoding strategies.

In contrast, the recent study of modulating metasurface properties in the time domain has not only led to the discovery of new physical phenomena but has also expanded the range of applications for metasurfaces^15–20^. Further, when combined with spatial manipulation, space-time-coding (STC) metasurface has been experimentally demonstrated, which is capable of simultaneously manipulating EM waves in both space and frequency domains^21^. Extensive studies have been conducted on time-varying and STC metasurfaces in recent years, resulting in many fascinating applications^22–37^, among them, Doppler cloaking^22^, harmonic beam steering^23–25^, the direction of arrival estimation^26,27^ and direct information modulation^31–34^. They have controlled the linear/nonlinear EM responses in a fixed polarization direction by phase manipulation. The recent utilization of anisotropic STC digital metasurfaces allowed for control over the beam and spectrum in two orthogonal polarization directions^38^, which can improve space utilization and channel capacity in wireless communications. However, the above studies lack the dimension of polarization manipulation.

The manipulation of polarization is crucial in various fields such as information multiplexing^39^, imaging^40^, and data storage^41^. While a large number of metasurface-based polarization converting approaches have been proposed^42–45^, the arbitrary rotation of linear polarization (LP) direction remains challenging. Significant efforts have been dedicated to achieving this goal^46–52^. For instance, a transmissive tunable rotator was proposed with a rotation range of 146°^47^. Nevertheless, those efforts have primarily focused on the fundamental frequency (linear frequency). In a recent development, time-domain digital metasurfaces^53,54^ have been employed for generating arbitrary polarizations at linear and nonlinear frequencies. However, these designs lack control over beam direction, and the theoretical conversion efficiency varies with the direction of polarization rotation in linear frequency.

**Fig. 1:**
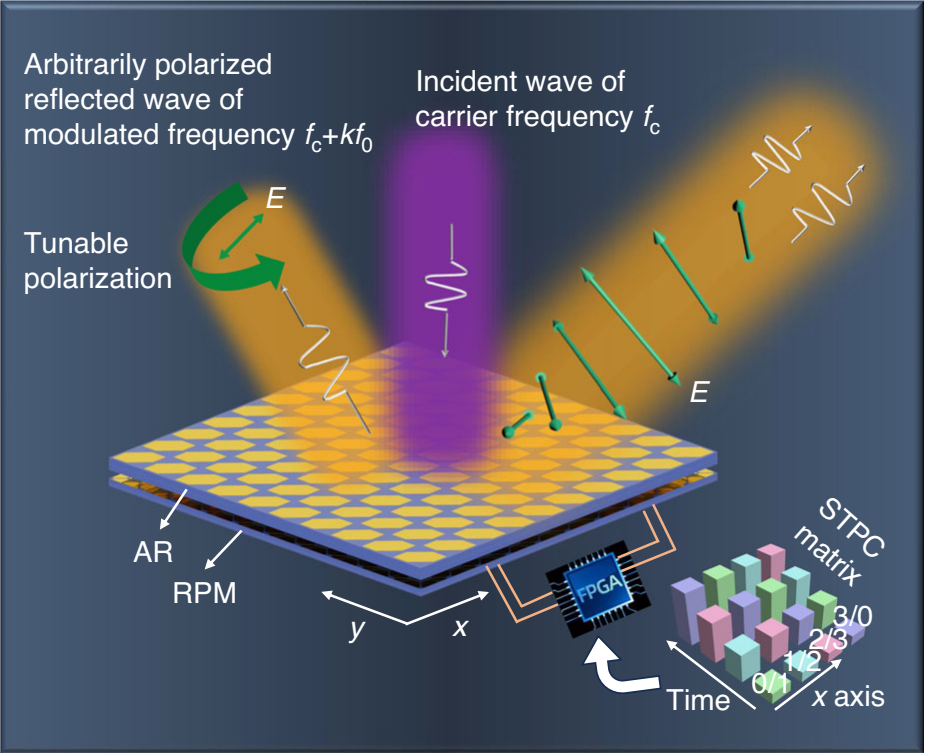
Conceptual illustration of the proposed design. The polarization direction, beam steering, and frequency of the reflected wave are controlled by the STPC matrix

Here we propose an approach that allows arbitrary manipulation of polarization direction and phase of the reflected wave. Further, we extend the STC theory to incorporate the dimension of polarization, which brings an additional degree of freedom for manipulating EM waves. To this aim, we design a staked programmable metasurface consisting of a reflective phase manipulation (RPM) structure and an anisotropic and reciprocal (AR) structure, as illustrated in Fig. 1. With the aid of a field-programmable gate array (FPGA) providing the space-time-polarization-coding (STPC) signal for the programmable metasurface, we achieve the real-time manipulation of polarization states, phase, and beam steering at linear and nonlinear frequencies. The proposed approach paves the way for multi-dimensional manipulation of EM waves.

## Results

### Theoretical concept and design

**Fig. 2:**
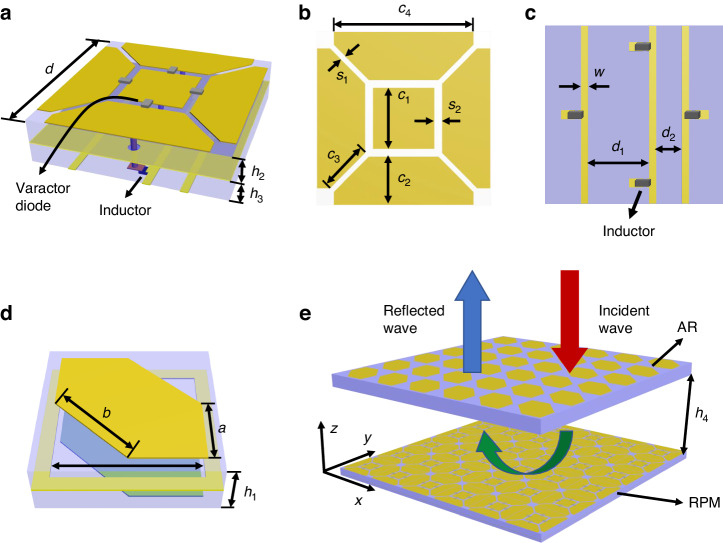
The design of the stacked programmable metasurface. **a** The element of the RPM structure, (**b**) the top and (**c**) the bottom metallic cladding of the RPM element. **d** The element of the AR structure. **e** The schematic of the stacked programmable metasurface. The dimensions are *d* = 20 mm, *a* = 8.8 mm, *b* = 12.45 mm, *h*_1_ = 6 mm, *h*_2_ = 2.5 mm, *h*_3_ = 2 mm, *c*_1_ = 7 mm, *c*_2_ = 5.5 mm, *c*_3_ = 5.69 mm, *c*_4_ = 16 mm, *s*_1_ = 0.6 mm, *s*_2_ = 0.9 mm, *d*_1_ = 6.3 mm, *d*_2_ = 2.9 mm, *w* = 0.8 mm, and *h*_4_ = 5 mm

Figure 2a–c depicts the RPM structure able to independently and continuously modulate the reflected phases in the *x*- and *y*-directions. Assuming that the amplitudes of co-polarized reflection are 1 under the *x*- and *y*-polarized waves, the Jones matrix of the RPM structure can be expressed as

1 $${R}_{{\rm{RPM}}}=\left[\begin{array}{cc}{R}_{xx} & {R}_{xy}\\ {R}_{yx} & {R}_{yy}\end{array}\right]=\left[\begin{array}{cc}{e}^{j{\varphi }_{xx}^{{\rm{RPM}}}} & 0\\ 0 & {e}^{j{\varphi }_{yy}^{{\rm{RPM}}}}\end{array}\right]={e}^{j\beta }\left[\begin{array}{cc}{e}^{-j\varDelta \varphi } & 0\\ 0 & {e}^{j\varDelta \varphi }\end{array}\right]$$

where the first and second subscripts represent the polarization direction of reflected and incident waves, respectively. ${\varphi }_{{xx}}^{{\rm{RPM}}}$ and ${\varphi }_{{yy}}^{{\rm{RPM}}}$ represent the phases of co-polarized reflection under the excitation of the *x*- and *y*-polarized waves, respectively. The half of the phase sum between *y*- and *x*-directions is denoted by *β* = (${\varphi }_{{yy}}^{{\rm{RPM}}}$ + ${\varphi }_{{xx}}^{{\rm{RPM}}}$)/2, and the half of the phase difference between *y*- and *x*-directions by ∆*φ* = (${\varphi }_{{yy}}^{{\rm{RPM}}}$-${\varphi }_{{xx}}^{{\rm{RPM}}}$)/2. To obtain independent and arbitrary control of polarization direction and phase, we introduce an AR structure shown in Fig. 2d, whose Jones matrix can be expressed as

2 $${T}_{{\rm{AR}}}=\left[\begin{array}{cc}{T}_{xx} & {T}_{xy}\\ {T}_{yx} & {T}_{yy}\end{array}\right]=\frac{\sqrt{2}}{2}\left[\begin{array}{cc}1 & j\\ j & 1\end{array}\right]$$

where the first and second subscripts represent the polarization direction of transmitted and incident waves, respectively. The RPM and AR structures are stacked up as depicted in Fig. 2e. An incident EM wave propagating along the -*z*-axis penetrates the AR structure first, followed by interactions with the RPM structure, and finally passes through the AR structure again and propagates in the +*z*-axis. Therefore, the Jones matrix of the whole system can be written as

3 $$\begin{array}{ll}{P}_{{\rm{whole}}}=\left[\begin{array}{cc}{\varGamma }_{xx} & {\varGamma }_{xy}\\ {\varGamma }_{yx} & {\varGamma }_{yy}\end{array}\right]={T}_{{\rm{AR}}}\cdot {R}_{{\rm{RPM}}}\cdot {T}_{{\rm{AR}}}\\\qquad\quad=\frac{{e}^{j\beta }}{2}\left[\begin{array}{cc}1 & j\\ j & 1\end{array}\right]\left[\begin{array}{cc}{e}^{-j\varDelta \varphi } & 0\\ 0 & {e}^{j\varDelta \varphi }\end{array}\right]\left[\begin{array}{cc}1 & j\\ j & 1\end{array}\right]\\\qquad\quad={e}^{j(\beta +\pi /2)}\left[\begin{array}{cc}-\,\sin \varDelta \varphi & \cos \varDelta \varphi \\ \cos \varDelta \varphi & \sin \varDelta \varphi \end{array}\right]\end{array}$$

Without the lack of generality, consider a *y*-polarized time-harmonic incident wave propagating along the -*z* direction. Its electric field can be described as

4 $${{\boldsymbol{E}}}_{y}^{i}=\hat{y}{e}^{j2\pi {f}_{c}t}={e}^{j2\pi {f}_{c}t}\left[\begin{array}{c}0\\ 1\end{array}\right]$$

where *f*_*c*_ indicates the frequency of the incident wave. The electric field of the reflected wave, denoted as ***E***^*r*^, can therefore be expressed as

5 $$\begin{array}{ll}{{\boldsymbol{E}}}^{r}={P}_{\text{whole}}\cdot {{\boldsymbol{E}}}_{y}^{i}\\\quad={e}^{j(\beta +\pi /2+2\pi {f}_{c}t)}\left[\begin{array}{c}\cos \varDelta \varphi \\ \sin \varDelta \varphi \end{array}\right]\\\quad=(\hat{{\boldsymbol{x}}}\,\cos \varDelta \varphi +\hat{{\boldsymbol{y}}}\,\sin \varDelta \varphi ){e}^{j(\beta +\pi /2+2\pi {f}_{c}t)}\end{array}$$

This equation reveals that the phase and polarization direction of the reflected wave are dependent on *β* and ∆*φ*, associated with the sum and the difference of ${\varphi }_{{xx}}^{{\rm{RPM}}}$ and ${\varphi }_{{yy}}^{{\rm{RPM}}}$, respectively. Since the RPM structure permits the independent and arbitrary control of ${\varphi }_{{xx}}^{{\rm{RPM}}}$ and ${\varphi }_{{yy}}^{{\rm{RPM}}}$, the phase and polarization direction of the reflected wave can be independently and arbitrarily manipulated. To obtain an arbitrary polarization angle, a wide tunable range exceeding 180° is required for both ${\varphi }_{{xx}}^{{\rm{RPM}}}$ and ${\varphi }_{{yy}}^{{\rm{RPM}}}$. More importantly, the conversion efficiency of arbitrary polarization direction is 100%.

To attain free control of polarization and phase at nonlinear frequency, we introduce time-varying phases ${\varphi }_{{xx}}^{{\rm{RPM}}}$(*t*) and ${\varphi }_{{yy}}^{{\rm{RPM}}}$(*t*) on the RPM structure. Here, we assume that the time-varying phases ${\varphi }_{{xx}}^{{\rm{RPM}}}$(*t*) and ${\varphi }_{{yy}}^{{\rm{RPM}}}$(*t*) are synchronous and have a period of *T*_0_, with a constant phase difference 2∆*φ* and a time-varying and periodic phase sum 2*β*(*t*). Thus, the Jones matrix of the whole system in the time domain can be written as

6 $$\begin{array}{ll}{P}_{{\rm{whole}}}(t)=\left[\begin{array}{cc}{\varGamma }_{xx}(t) & {\varGamma }_{xy}(t)\\ {\varGamma }_{yx}(t) & {\varGamma }_{yy}(t)\end{array}\right]\\\qquad\qquad={e}^{j(\beta (t)+\pi /2)}\left[\begin{array}{cc}-\,\sin \varDelta \varphi & \cos \varDelta \varphi \\ \cos \varDelta \varphi & \sin \varDelta \varphi \end{array}\right]\end{array}$$

It can be seen from Eq. (6) that for *y*-polarized wave excitation, the co-polarized reflection coefficient *Γ*_*yy*_(*t*) is

7 $${\varGamma }_{yy}(t)={e}^{j(\beta (t)+\pi /2)}\sin \varDelta \varphi$$

and the cross-polarized reflection coefficient *Γ*_*xy*_(*t*) is

8 $${\varGamma }_{xy}(t)={e}^{j(\beta (t)+\pi /2)}\cos \varDelta \varphi$$

Obviously, the reflection phase is a time-varying periodic signal *β*(*t*). According to the Fourier transform, the spectrum of the reflected wave will transform from linear frequency to nonlinear frequencies^18^. The phase and polarization direction at the nonlinear frequency depends on initial phase *β*(*t* = 0) and ∆*φ*. To simultaneously control the polarization direction, the spectrum, and the beam pointing of the reflected wave, we introduce the STPC strategy. Here, we consider an STPC metasurface composed of a 2D array of *M* × *N* elements. According to the developed theory of STC metasurfaces^21^, the far-field electric field scattered by the STPC metasurface can be extended as

9 $${\boldsymbol{E}}(\theta ,\varphi ,t)=\mathop{\sum }\limits_{q=1}^{N}\mathop{\sum }\limits_{p=1}^{M}{{f}}_{pq}(\theta ,\varphi )\Big[{\varGamma }_{yy}^{pq}(t)\hat{{\boldsymbol{y}}}+{\varGamma }_{xy}^{pq}(t)\hat{{\boldsymbol{x}}}\Big]$$

and

10 $$\begin{array}{l}{{f}}_{pq}(\theta ,\varphi )={E}_{pq}(\theta ,\varphi )\exp \Big\{j\frac{2\pi }{{\lambda }_{c}}\Big[(p-1){d}_{x}\,\sin \theta \,\cos \varphi \\\qquad\qquad+\,(q-1){d}_{y}\,\sin \theta \,\sin \varphi \Big]\Big\}\end{array}$$

where *E*_*pq*_(*θ*, *φ*) denotes the scattering pattern of the (*p*, *q*)th coding element at frequency *f*_c_, *θ,* and *φ* represent the elevation and azimuth angles, respectively, *λ*_c_ = c/*f*_c_ denotes the wavelength of the incident wave, and *dx* and *dy* are the periods of the coding elements along the *x* and *y*-directions, respectively. ${\varGamma }_{{yy}}^{{pq}}$(*t*) and ${\varGamma }_{{xy}}^{{pq}}$(*t*) are the co- and cross-polarized time-modulated reflection coefficients of the (*p*, *q*)th coding element, respectively. Assuming the modulation frequency *f*_0_ = 1/*T*_0_ is much smaller than the frequency *f*_*c*_ of the incident wave, and applying Fourier series expansion of the reflection coefficient ${\varGamma }_{{yy}}^{{pq}}$(*t*) and ${\varGamma }_{{xy}}^{{pq}}$(*t*), the scattered far fields at the *k*-th harmonic frequency *f*_*c*_ + *kf*_0_ can be written as

11 $${{\boldsymbol{E}}}_{k}(\theta ,\varphi )=\mathop{\sum }\limits_{q=1}^{N}\mathop{\sum }\limits_{p=1}^{M}{{f}}_{pq}(\theta ,\varphi )\left[{a}_{yy}^{pq,k}\hat{{\boldsymbol{y}}}+{a}_{xy}^{pq,k}\hat{{\boldsymbol{x}}}\right]$$

where the wavelength of the *k*-*th* order harmonics is *λ*_*k*_ = *c*/(*f*_*c*_ + *kf*_0_). ${a}_{{yy}}^{{pq},{k}}$ is the Fourier expansion of the reflection coefficient ${\Gamma }_{{yy}}^{{pq}}$(*t*), and it is given by^21^

12 $${a}_{yy}^{pq,k}=\mathop{\sum }\limits_{n=1}^{L}\frac{{\varGamma }_{n}^{pq}}{\pi k}\sin \left(\frac{\pi k}{L}\right)\exp \left[\frac{-j\pi k(2n-1)}{L}+\pi /2\right]$$

where ${\varGamma}_{n}^{{pq}}$ denotes the co-polarized reflection coefficient of the *n*-th time-coding sequence with the length of *L*. ${a}_{{xy}}^{{pq},{k}}$ is the Fourier expansion of ${\varGamma }_{{xy}}^{{pq}}$(*t*), according to Eqs. (7) and (8) it is given

13 $${a}_{xy}^{pq,k}={a}_{yy}^{pq,k}\,\tan \varDelta \varphi$$

Equation (11) provides an insight into the manipulation capabilities of the proposed design. It highlights that the polarization, the beam pointing, and the spectrum of the reflected wave can be modulated simultaneously.

The elements of RPM and AR structure are depicted in Fig. 2a–c, d, respectively. The RPM element (see Fig. 2a–c) consists of three metal layers and two F4B dielectric substrates (*ϵ*_*r*_ = 2.4 and tan *δ* = 0.001). The top layer is patterned with a square patch and two pairs of symmetrically placed polygonal patches, as shown in Fig. 2b. A total of four varactor diodes (Skyworks SMV1405-079LF) are soldered at the gaps between the rectangular patch and polygonal patches. Specifically, the square patch and each polygonal patch are connected to the middle metal ground and the feeding line located in the bottom layer through metalized vias, respectively, allowing separate direct current (DC) biasing voltages to be applied in both the *x* and *y*-directions. The middle metal layer is the radio frequency (RF) ground that acts as a reflector. To isolate the RF signals in the top layer from DC biasing in the bottom layer, as shown in Fig. 2c, four inductors (LQW15AN43NJ00) are soldered between the feeding line and the metalized via. The element of the AR structure depicted in Fig. 2d is composed of three metal layers and two F4B dielectric substrates (*ϵ*_*r*_ = 2.6 and tan *δ* = 0.001). The top and bottom metal patches are identical.

**Fig. 3:**
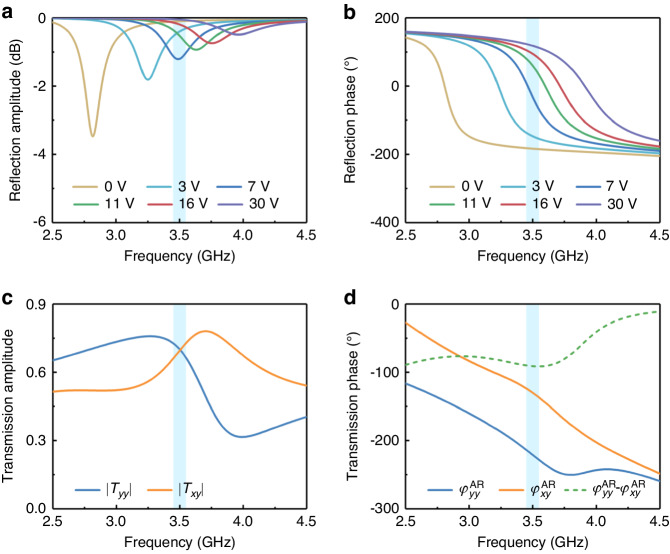
The simulated results of the RPM and AR structure under y-polarized wave excitation. The simulated reflection (**a**) amplitudes and (**b**) phases of the RPM structure. The simulated transmission (**c**) amplitudes and (**d**) phases of the AR structure. Due to the symmetry of the RPM and AR structure, only the simulated results under the excitation of a *y*-polarized wave are presented

To assess that the designed RPM and AR structures satisfy the Jones matrices described in Eqs. (1) and (2), respectively, full-wave simulations of the elements were conducted in CST Microwave Studio. The simulated reflection amplitudes and phases of the RPM structure under *y*-polarized wave excitation are depicted in Fig. 3a, b, respectively. The simulation revealed a phase range of 308° at 3.5 GHz with reflection amplitudes |*R*_*yy*_| of above −1.5 dB when the biasing voltage ranged from 0 V to 30 V. It should be noted that the phase range could potentially be improved by using a varactor diode with a larger capacitance range or by using a substrate with lower thickness. Due to the symmetry of the RPM structure, only the results for *y*-polarized wave incidence are presented in this paper. This study demonstrates that the RPM structure can be well characterized by Eq. (1) with the assumption of the reflected amplitudes for most cases except for that with a biasing voltage around 7 V which causes a reduced amplitude. The mapping relationship between the reflected phases of the RPM structure and the control voltages can be found in Note S1 of Supplementary Information.

Additionally, under the excitations of *y*-polarized waves, the simulated transmission amplitudes and phases of the AR structure are shown in Fig. 3c, d, respectively. The transmission amplitude |*T*_*yy*_| and |*T*_*xy*_| remain ~0.7 over a frequency range centered at 3.5 GHz. It is also worth noting that the transmission phase difference between the co- and cross-polarized components is ~90°. Likewise, due to the symmetry of the AS structure, only the results under the excitation of a *y*-polarized wave are presented in this paper. Therefore, the transmission response of the AR structure is well characterized by Eq. (2).

### Manipulation of polarization and phase at the linear frequency

**Fig. 4:**
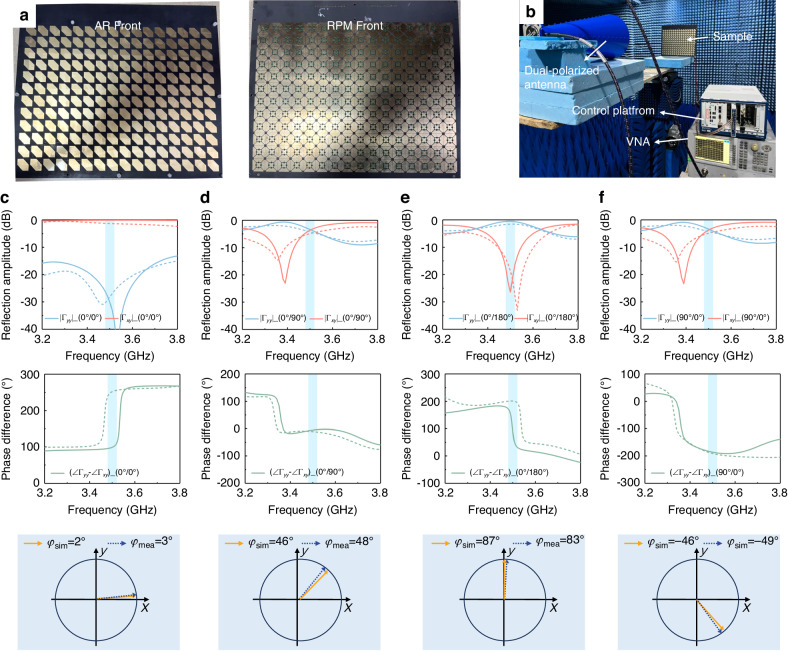
The simulated and measured results for different phase combinations. **a** Photograph of the fabricated metasurface sample. **b** Experimental setup and the environment in an anechoic chamber. Under *y*-polarized wave excitation, (**c**–**f**) the simulated and measured amplitudes (upper panel), phase differences ($\angle$*Γ*_*yy*_-$\angle$*Γ*_*xy*_) (middle panel), and polarization direction (bottom panel) for voltage-controlled phase combinations in the *x*-/*y*-directions of 0°/0°, 0°/90°, 0°/180°, and 90°/0°, respectively, where the solid lines and the dashed lines indicate the simulated and measured results, respectively

To evaluate the overall performance of the staked programmable metasurface, a prototype was simulated, fabricated, and measured. Figure 4a showcases the fabricated sample, which consists of 16 × 12 elements with an overall size of 300 × 290 mm^2^. Figure 4b depicts the measurement of the reflection coefficient. The metasurface’s ability to rotate polarization was first evaluated. The +*x* direction is defined as 0°, and the counterclockwise direction represents the positive direction of polarization. The simulated and measured results under different phase combinations for y-polarized wave incidence are presented in Fig. 4c–f. It is evident that the simulated and measured phases mostly coincide with each other except for phase differences ($\angle$*Γ*_*yy*_-$\angle$*Γ*_*xy*_) in the cases of 0°/0° and 0°/180°. However, since the co-polarized amplitude in the case of 0°/0° and the cross-polarized amplitude in the case of 0°/180° are negligibly low, the values of the phase differences have a negligible effect on the polarization direction. According to Eq. (5), the polarization directions of the reflection waves under different phase combinations depicted in Fig. 4c–f were theoretically calculated to be 0°, 45°, 90°, and −45°, respectively. The simulated polarization angle *φ*_sim_ and measured polarization angle *φ*_mea_, listed in the bottom panel of Fig. 4c–f, were found to largely agree with the theoretical calculations. The slight discrepancies between the simulated and theoretical polarization angle may arise from the imbalanced reflection amplitudes of the RPM structure in the *x*- and *y*-directions, while the slight discrepancies between the simulated and measured polarization angle can be attributed to imperfect fabrication processing and measurement deviations. Further, the polarization angle is not limited to only discrete states. Since the reflection phase varies continuously with the biasing voltage and the phase difference ∆*φ* is greater than 180°, the proposed design is capable of rotating polarization to an arbitrary direction.

**Fig. 5:**
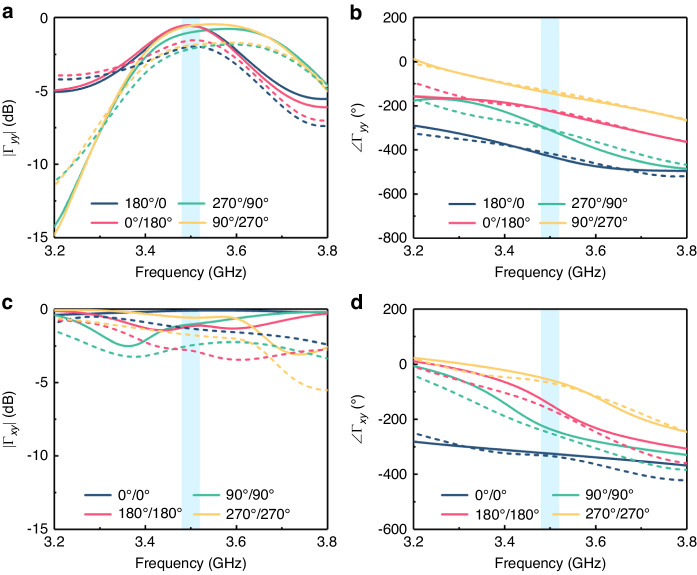
Phase manipulation of 0°- and 90°-polarized reflection waves under y-polarized wave excitation. The simulated and measured (**a**) amplitudes and (**b**) phases of the 0°-polarized reflection wave in case of phase combination of 0°/0°, 90°/90°, 180°/180°, and 270°/270°. The simulated and measured (**c**) amplitudes and (**d**) phases of the 90°-polarized reflection wave in case of phase combination of 180°/0°, 270°/90°, 0°/180°, and 90°/270°. The solid lines and the dashed lines indicate the simulated and measured results, respectively

Next, the phase-modulating capability in polarization rotation directions was evaluated. Here, we showcase four phase states (the interval between the adjacent phase is 90°) of the 0°- and 90°-polarized reflection waves. The four phase states of 45 and −45°-polarized reflection waves can be found in Note S2 of Supplementary Information. According to Eq. (5), the four phase states of the 0°-polarized reflection wave can be obtained by phase combinations of 0°/0°, 90°/90°, 180°/180°, and 270°/270°, Similarly, the four phase states of the 90°-polarized reflection wave can be generated by using phase combination of 180°/0°, 270°/90°, 0°/180°, and 90°/270°. The simulated and measured results with different phase combinations are presented in Fig. 5. The simulated/measured amplitudes were above −1.2/−2.8 dB at 3.5 GHz in all cases, and the measured phases exhibited good agreement with the simulated ones. Based on the above outcomes, it is evident that the proposed programmable metasurface can independently manipulate polarization and phase at a linear frequency.

### Manipulation of polarization and beam at the linear frequency

Since the designed programmable metasurface exhibits the capability to independently manipulate polarization and phase at the linear frequency, we can control beam deflection, as well as polarization manipulation. Note, the polarization direction is defined in the plane orthogonal to the propagation direction of the beam. The 0°-, 45°- and 90°-polarized reflection waves were engineered as examples, and 2-bit phase coding strategies were employed, where RPM structure’s reflection phases (${\varphi }_{{xx}}^{{\rm{RPM}}}$ and ${\varphi }_{{yy}}^{{\rm{RPM}}}$) of 0°, 90°, 180°, and 270° were denoted as the code states 0, 1, 2, and 3 respectively. Additionally, the number before and after the slash symbol (/) represents the code state in the *x*- and *y*-directions, respectively.

**Fig. 6:**
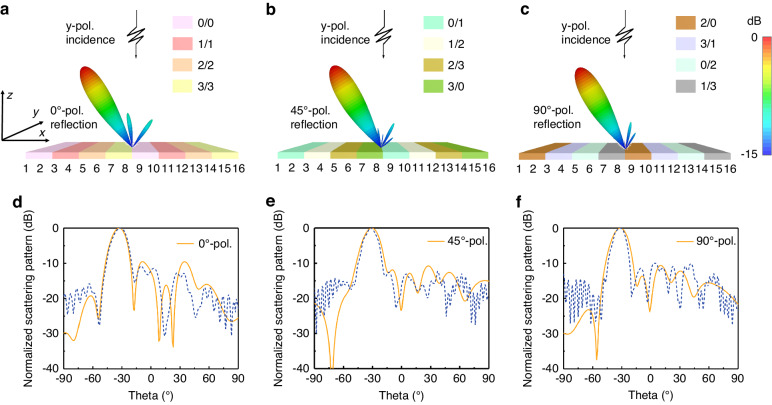
The normalized far-field scattering patterns under y-polarized wave excitations. The simulated normalized 3D scattering patterns of (**a**) 0°-, (**b**) 45°-, (**c**) 90°-polarized reflection at 3.5 GHz. The simulated and measured normalized 2D scattering patterns of (**d**) 0°-, (**e**) 45°-, (**f**) 90°-polarized reflection at 3.5 GHz. The polarization is defined in the plane orthogonal to the propagating direction of the beam. The solid lines and the dashed lines indicate the simulated and measured results, respectively

The programmable metasurface array, composed of 16 × 12 meta-atoms, was encoded accordingly (Fig. 6a–c), with the phase coding of each meta-atom represented by distinct colored blocks. Without loss of generality, we consider a 1D spatial coding scenario, where each column of meta-atoms along the *y*-direction featured the same coding pattern. For the 0°-polarized reflection wave, a periodic space-polarization-coding (SPC) sequence of 0/0-0/0-1/1-1/1-2/2-2/2-3/3-3/3… was applied onto the programmable metasurface array. Based on Eq. (9), the resulting deflecting angle of the beam with respect to the *z*-axis was calculated to be about 32.4°. As depicted in Fig. 6a, the simulated 3D far-field scattering pattern showcased a directive beam with a deflecting angle of ~32° for the 0°-polarized reflection wave. Similarly, a periodic SPC sequence for the 45°-polarized reflection wave was 0/1-0/1-1/2-1/2-2/3-2/3-3/0-3/0… (Fig. 6b), and a periodic SPC sequence for the 90°-polarized reflection wave followed the pattern 2/0-2/0-3/1-3/1-0/2-0/2-1/3-1/3… (Fig. 6c). Corresponding to these SPC sequences, Fig. 6d–f display the simulated and measured results of the normalized 2D scattering patterns (see more details of measurement in the Methods and Fig. S2 in the Supplementary Information). The simulated and measured results exhibit good agreement with only minor discrepancies that may come from imperfections in the fabrication and measurement. Comparing with the scattering patterns of the metal plate of the same size as the metasurface, the simulated/measured gain of the 0°-, 45°-, and 90°-polarized waves exhibit reductions of ~3.1/3.9 dB, 3.4/4 dB, and 2.9/3.8 dB, respectively. By altering the SPC scheme, the proposed programmable metasurface can achieve other beam deflection and polarization angles. Additional beam steering angles at the linear frequency are provided in Supplementary Information Note S4. The measured results demonstrate the feasibility of the designed programmable metasurface in simultaneously controlling polarization and beam angle of reflection wave at the fundamental frequency.

### Manipulation of polarization and beam at the nonlinear frequency

**Fig. 7:**
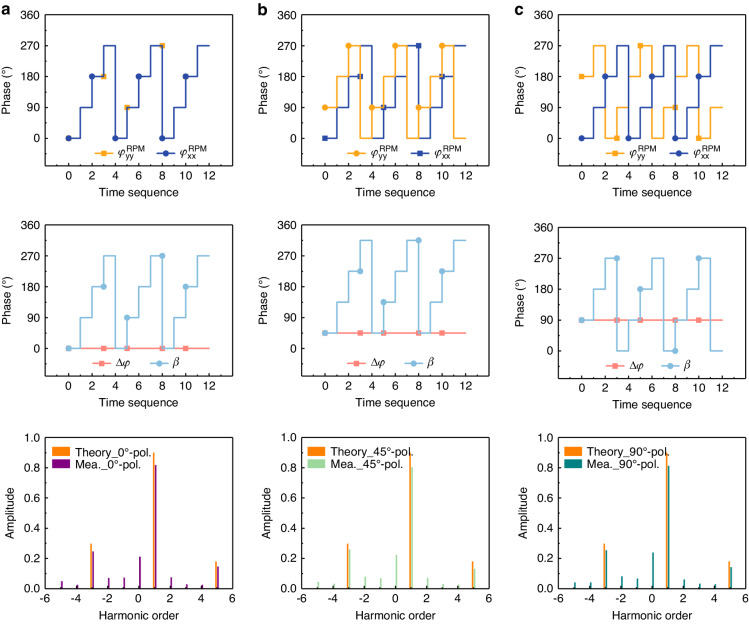
The TPC sequences and spectral intensity of harmonics. The TPC sequences of ${\varphi }_{{xx}}^{{\rm{RPM}}}$(*t*) and ${\varphi }_{{yy}}^{{\rm{RPM}}}$(*t*) (upper panel), their corresponding ∆*φ*(*t*) and *β*(*t*) (middle panel), as well as their corresponding theoretical and measured spectral intensity of harmonics (bottom panel) for the (**a**) 0°-, (**b**) 45°-, and (**c**) 90°-polarized reflection waves, where the length of the TPC sequence is *L* = 4, and modulation frequency is *f*_0_ = 100 kHz. The solid lines and the dashed lines indicate the simulated and measured results, respectively

In the previous subsection, independent control of polarization and beam at the linear frequency was successfully implemented. To extend this function to nonlinear frequency, synchronously time-varying phases ${\varphi }_{{xx}}^{{\rm{RPM}}}$(*t*) and ${\varphi }_{{yy}}^{{\rm{RPM}}}$(*t*) were introduced. According to Eq. (5), the polarization and phase at nonlinear frequency depend on ∆*φ* and *β*(*t* = 0), respectively, which are easily controlled by biasing voltages. We first investigate the programmable metasurface’s ability to control polarization at nonlinear frequencies and take +1st-order harmonic conversion for 0°, 45°, and 90°-polarized reflection waves as examples. According to Eq. (11), to achieve complete conversion of the +1st-order harmonic, the reflected phase range within a single time period reaches 360°, with a continuous modulation of phase, which requires a very high modulation rate for the control circuit. Moreover, given the constraints of ${\varphi }_{{xx}}^{{\rm{RPM}}}$ and ${\varphi }_{{yy}}^{{\rm{RPM}}}$ within the range of 308°, we have adopted 2-bit time-coding strategies. The time-polarization-coding (TPC) sequences for ${\varphi }_{{xx}}^{{\rm{RPM}}}$(*t*) and ${\varphi }_{{yy}}^{{\rm{RPM}}}$(*t*), as well as ∆*φ*(*t*) and *β*(*t*) values are presented in Fig. 7a–c, where the length of the TPC sequence (*L*) is set to 4, and the modulation frequency (*f*_0_) is 100 kHz. It can be observed that ∆*φ*(*t*) remains constant in all cases, while *β*(*t*) is a 2-bit TPC sequence. The theoretical and measured spectral intensity distributions of harmonics are depicted in the bottom panel of Fig. 7. The measured spectrum intensities agree well with the theoretical predictions at most harmonics except at the 0th-order harmonic (at 3.5 GHz) where the measured amplitude is considerably higher than zero. This discrepancy may be attributed to the fact that the incident wave cannot fully interact with the programmable metasurface. Additionally, the weak spectral energy at other orders is detected in the measurement while the theoretical energy is zero. This could be caused by the adjacent TPC states being not exactly 90°. Evidently, the proposed programmable metasurface enables efficient frequency conversion from linear frequency to nonlinear frequencies, simultaneously providing control over the polarization at the nonlinear frequency.

**Fig. 8:**
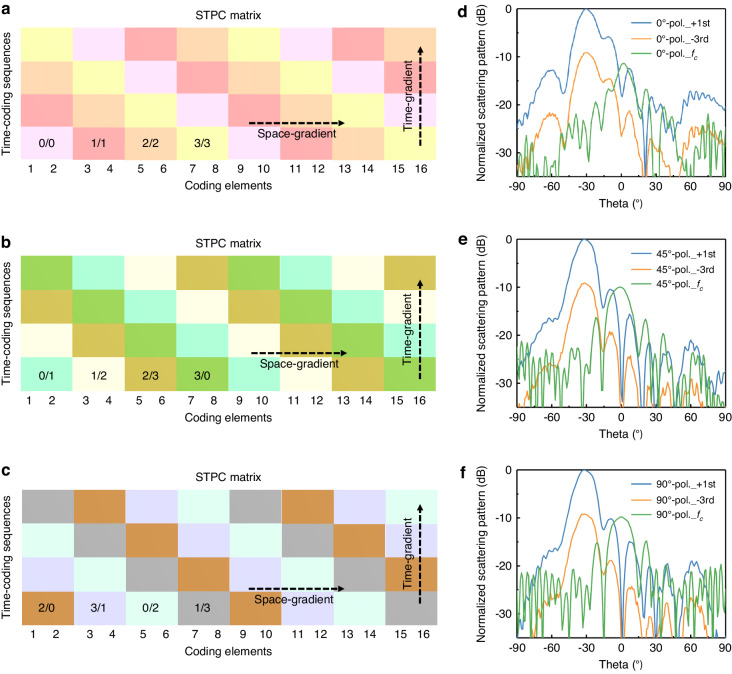
2D STPC matrices and measured normalized far-field scattering patterns. 2D STPC matrices of dimension 16 × 4 capable of generating the (**a**) 0°-, (**b**) 45°-, and (**c**) 90°-polarized reflection wave, where distinct colored blocks represented different phase coding. The measured normalized far-field scattering patterns of (**d**) 0°-, (**e**) 45°-, and (**f**) 90°-polarized reflection waves at the 0th-, +1st- and -3rd-order harmonics. The polarization is defined in the plane orthogonal to the propagating direction of the beam

Finally, to evaluate the simultaneous control over polarization and beam at nonlinear frequencies, we introduce spatial distributions of 2-bit initial phases along the *x*-direction on the basis of the TPC scheme shown in Fig. 7. 2D STPC matrices of dimension 16 × 4 capable of generating the 0°-, 45°- and 90°-polarized reflection waves are shown in Fig. 8a–c, respectively. The measured results of corresponding polarization direction at the 0th-, +1st- and -3rd-order harmonics wave are depicted in Fig. 8d–e (see more details of measurement in Methods and Fig. S3 in the Supplementary Information). Notably, the maximum scattering directivity for the 0th-order harmonic exhibits a significant drop compared to that of the +1st-order, indicating an efficient spectrum conversion. By using Eq. (11), the deflection angle of the beam at the +1st- and -3rd-order harmonic wave can be calculated as 32.391° and 32.395°, respectively, which agree well with the measured beam deflection angles of ~32° for the 0°-, 45°- and 90°-polarized reflection waves at +1st- and -3rd-order harmonics. Other orders were not included in the analysis due to their low energy levels. Comparing with the scattering patterns of the metal plate with an equivalent size to the metasurface, the measured gain for the 1st-order harmonic decreases by ~6.7 dB, 7.6 dB, and 7.1 dB for the 0°-, 45°-, 90°-polarized waves, respectively. The beam steering of other angles at the nonlinear frequency is provided in Supplementary Information Note S5. Our study demonstrates consistent agreement between the theoretical predictions and measurements of the spectral distributions, their respective beam deflections, and polarization rotation at nonlinear frequencies. This final study demonstrates that the proposed programmable metasurface possesses the ability to achieve independent control of polarization and beam at nonlinear frequencies, thus highlighting its potential for various practical applications.

## Discussion

In summary, we proposed an approach that can arbitrarily manipulate the polarization direction and phase of the reflected wave in linear and nonlinear ways. As a proof of principle, a stacked programmable metasurface was designed, simulated, and measured. By separately modulating the control voltage of varactor diodes oriented at the *x*- and *y*-directions, we achieved a high polarization rotation range covering the entire azimuthal angles at linear and nonlinear frequency, as well as adjustable phase. Further, integrating STC theory with our design facilitates simultaneous manipulations of the polarization state and beam steering at linear and nonlinear frequencies. The experimental outcomes showcase consistency with theoretical predictions and simulation, validating the feasibility of our proposed approach. Our approach opens new avenues for the multi-dimensional manipulation of EM waves. Although the designed programmable metasurface operates in the microwave band, the proposed concept can be extended into terahertz and even optical domains, which have promising applications in radar, imaging, and wireless communications.

## Materials and methods

### Experimental setup

The measurement of the reflection coefficient was performed in a microwave anechoic chamber, as depicted in Fig. 4b. A dual-polarized horn antenna was connected to the vector network analyzer (VNA, Agilent N5230C), and was positioned in the front of the sample. A control platform (PXIe1092, NI Corp.) including an FPGA module is employed to provide control signals for the programmable metasurface in real time, where each column shares a consistent control voltage. A same-sized metallic sheet was used as a reference.

The measurement of the far-field scattering pattern at linear frequency is depicted in Fig. S2 of Supplementary Information. A pair of high-gain horn antennas, one acting as the transmitting antenna and the other as the receiving antenna, were connected to the VNA. The transmitting horn antenna was positioned in the front of the sample and emitted a *y*-polarized wave. Both the programmable metasurface and transmitting horn antenna are positioned on a turntable that can automatically rotate by 360°. Another horn antenna whose polarization can be adjusted as required was employed to receive the scattering field at different azimuthal angles. The control platform offered constant signals for the programmable metasurface.

The measurement of the programmable metasurface at nonlinear frequencies is shown in Fig. S3 of Supplementary Information. The transmitting horn antenna was connected to a signal generator (Keysight E8267D) and emitted an EM wave with a frequency of 3.5 GHz. The receiving antenna was connected to the VNA and received the harmonic wave. Moreover, the control platform offered time-varying periodic signals with modulation period *T*_0_ for the programmable metasurface.

### Supplementary information

Supplementary information
