# Supplementary material for: Arbitrarily rotating polarization direction and manipulating phases in linear and nonlinear ways using programmable metasurface
Source: Light Sci Appl. 2024 Jul 18;13:172. doi: 10.1038/s41377-024-01513-2 (PMC11258343; doi:10.1038/s41377-024-01513-2)
Supplement: Supplementary file 1 — Supplementary information [file 41377_2024_1513_MOESM1_ESM.docx]

Supplementary Information

**Arbitrarily rotating polarization direction and manipulating phases in linear and nonlinear ways using programmable metasurface**

Wei Liu ^1,2^, Si Ran Wang ^1,2^, Jun Yan Dai ^1,2,3,*^, Lei Zhang ^1,2^, Qiao Chen ^4,*^, Qiang Cheng ^1,2,3,*^,and Tie Jun Cui^1,2,3,*^

*^1^ State Key Laboratory of Millimeter Waves, Southeast University, Nanjing 210096, China*

*^2^ Institute of Electromagnetic Space, Southeast University, Nanjing 210096, China*

*^3^ Frontiers Science Center for Mobile Information Communication and Security, Southeast University, Nanjing 210096, China*

*^4^ Electrical Engineering Department, Chalmers University of Technology, Gothenburg 41258, Sweden.*

*These authors contributed equally: Wei Liu, Si Ran Wang, Jun Yan Dai*

E-mail: junyand@seu.edu.cn, qiaoc@chalmers.se, qiangcheng@seu.edu.cn and [tjcui@seu.edu.cn](mailto:tjcui@seu.edu.cn)

**Note S1. The mapping relationship between the reflection phases and the control voltages**

**Table S1.** The mapping relationship between the reflection phases ($\varphi_{xx}^{\mathrm{RPM}}$ and $\varphi_{yy}^{\mathrm{RPM}}$) of the RPM structure and the control voltages.

| Control Voltage (V) | 0 | 3 | 5.2 | 7 | 8.9 | 11 | 15.2 | 30 |
| --- | --- | --- | --- | --- | --- | --- | --- | --- |
| $\varphi_{xx}^{\mathrm{RPM}}$=$\varphi_{yy}^{\mathrm{RPM}}$ (Deg.) | 0° | 39° | 90° | 151° | 180° | 243° | 270° | 308° |

**Note S2. The four phase states of 45°- and -45°-polarized reflection waves**

According to Equation (5), the four phase states of the 45°-polarized reflection wave can be obtained by phase combination of 0°/90°, 90°/180°, 180°/270°, and 270°/0°, and the four phase states of the -45°-polarized reflection wave can be obtained by phase combination of 90°/0°, 180°/90°, 270°/180°, and 0°/270°. The simulated and measured results with different phase combinations are presented in Figure S1. The measured amplitudes are above -2.8 dB at 3.5 GHz in all cases, and the phase differences between the adjacent phase combinations are approximately 90°. The measured results are in agreement with the simulated results.


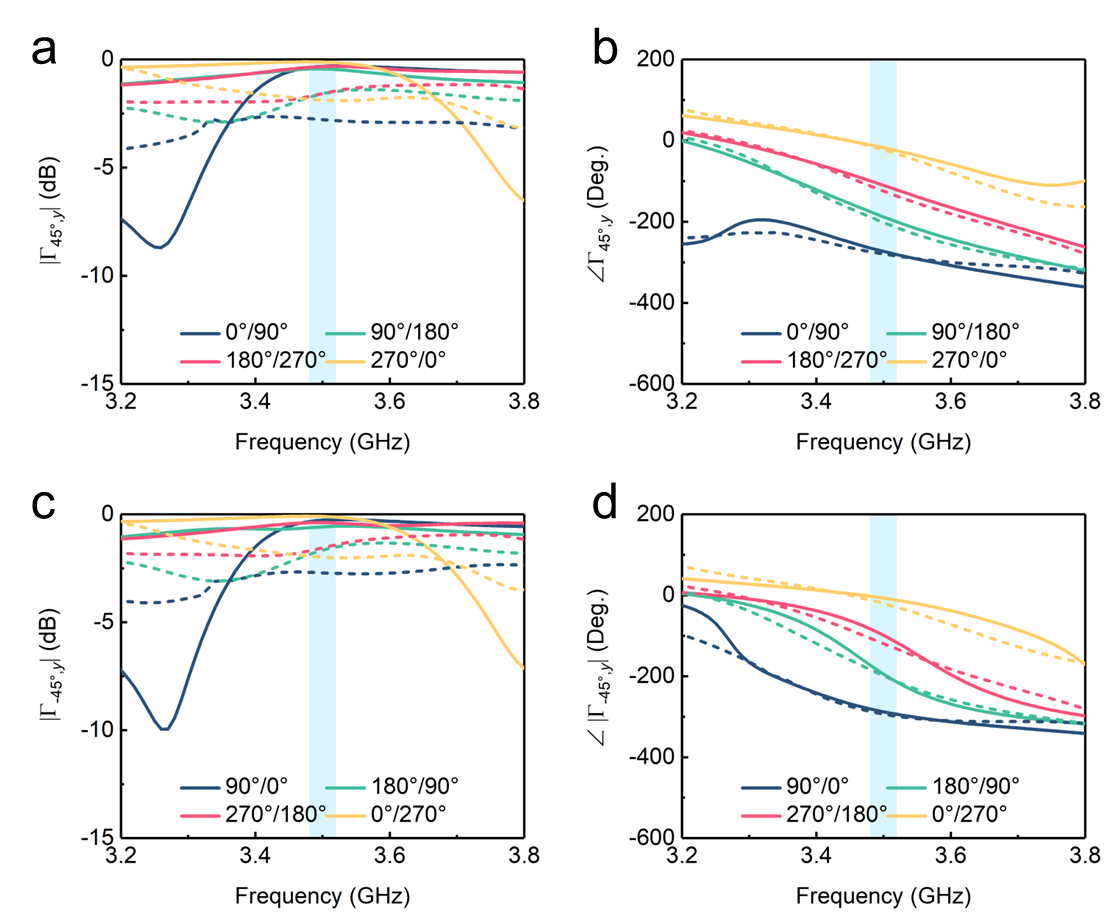


**Fig. S1.** Phase manipulation of 45°- and -45°-polarized reflection waves. Under *y*-polarized wave excitation, The simulated and measured (a) amplitudes and (b) phases of the 45°-polarized reflection wave in case of phase combination of 0°/90°, 90°/180°, 180°/270°, and 270°/0°. The simulated and measured (c) amplitudes and (d) phases of the -45°-polarized reflection wave in case of phase combination of 90°/0°, 180°/90°, 270°/180°, and 0°/270°. The solid lines and the dashed lines indicate the simulated and measured results, respectively.

**Note S3. The measurement setup**

The measurement of the far-field scattering pattern at the linear frequency and the measurement setup at the nonlinear frequencies are illustrated in Figures S2 and S3, respectively.


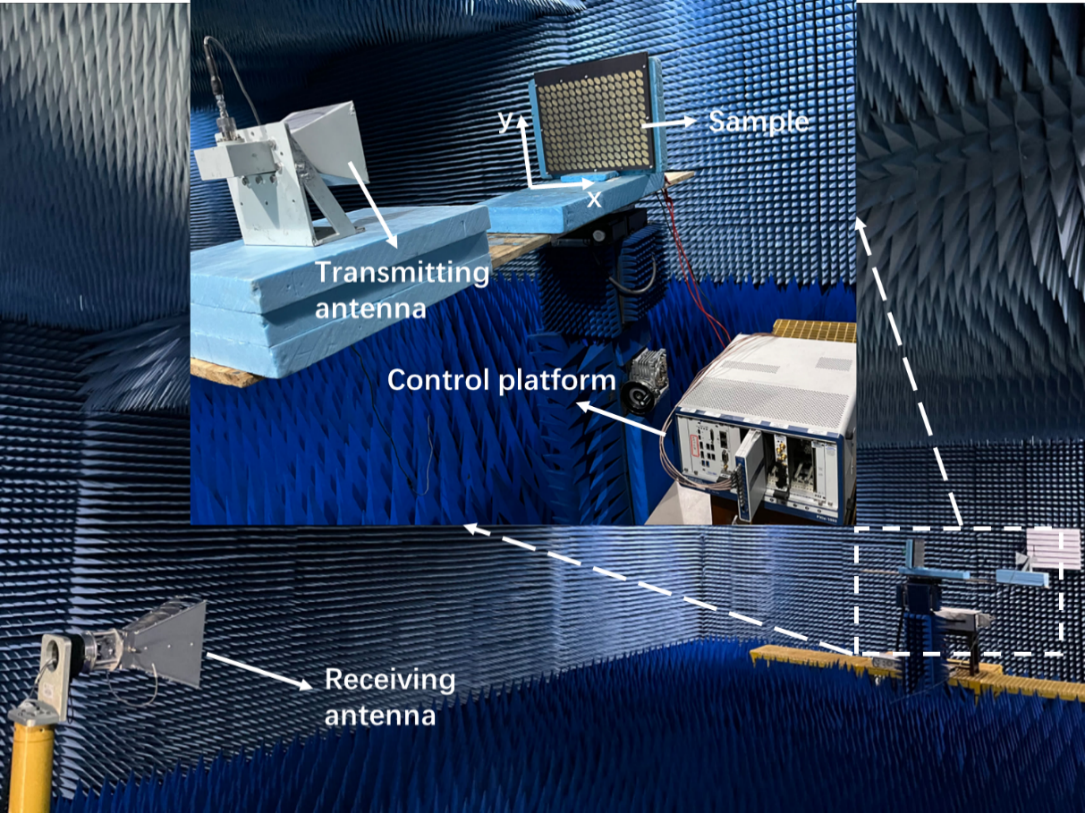


**Fig. S2.** The measurement of the far-field scattering pattern at the linear frequency


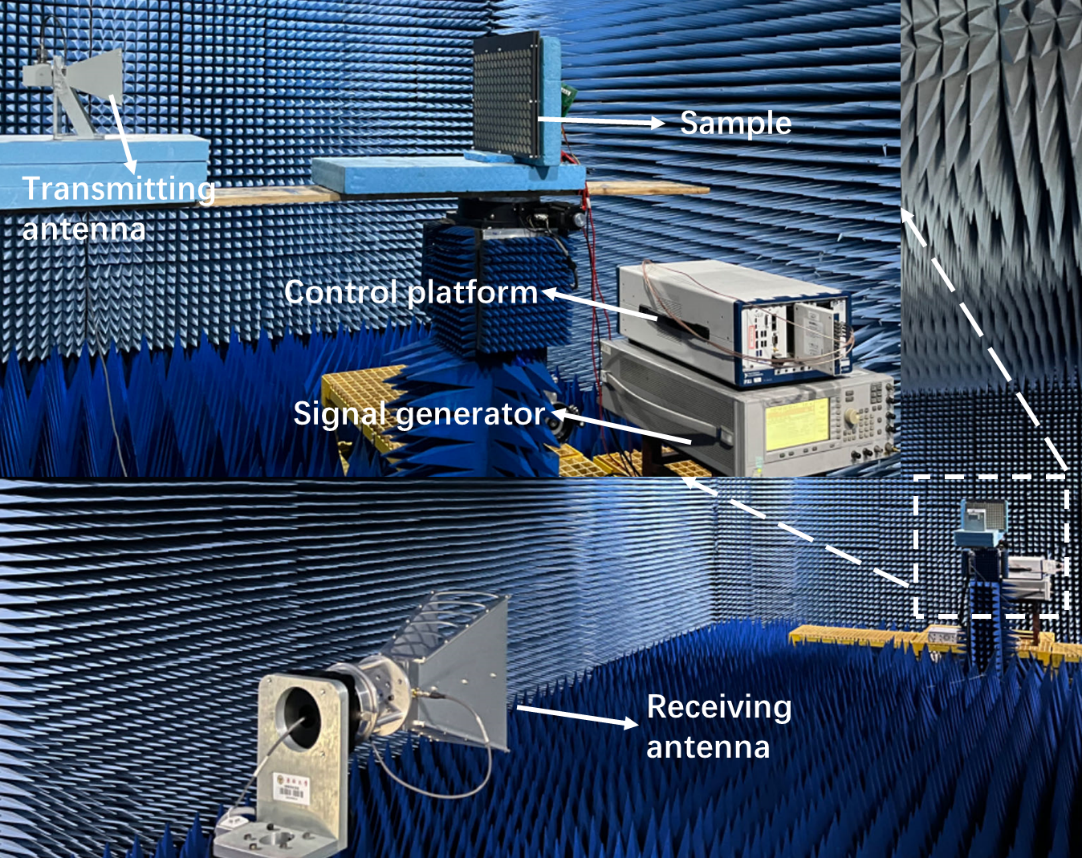


**Fig. S3.** The measurement setup at the nonlinear frequencies

**Note S4. Manipulation of polarization and beam at the linear frequency.**

For the 0°-polarized reflection wave, a SPC sequence of 3/3-3/3-3/3-2/2-2/2-2/2-1/1-1/1-1/1-0/0-0/0-0/0-3/3-3/3-3/3-2/2 is applied onto the programmable metasurface array, and the SPC sequences for the 45°- and 90°-polarized reflection waves followed patterns of 3/0-3/0-3/0-2/3-2/3-2/3-1/2-1/2-1/2-0/1-0/1-0/1-3/0-3/0-3/0-2/3 and 1/3-1/3-1/3-0/2-0/2-0/2-3/1-3/1-3/1-2/0-2/0-2/0-1/3-1/3-1/3-0/2, respectively. Based on Equation (9), the deflecting angle of the beam for different polarized waves are calculated as about 20.9°. Corresponding to these SPC sequences, Figures S4(a-c) show the simulated and measured results of the normalized 2D scattering patterns. In all cases, the deflection angles are about 21°. The simulated and measured results exhibit good agreement with only minor discrepancies.


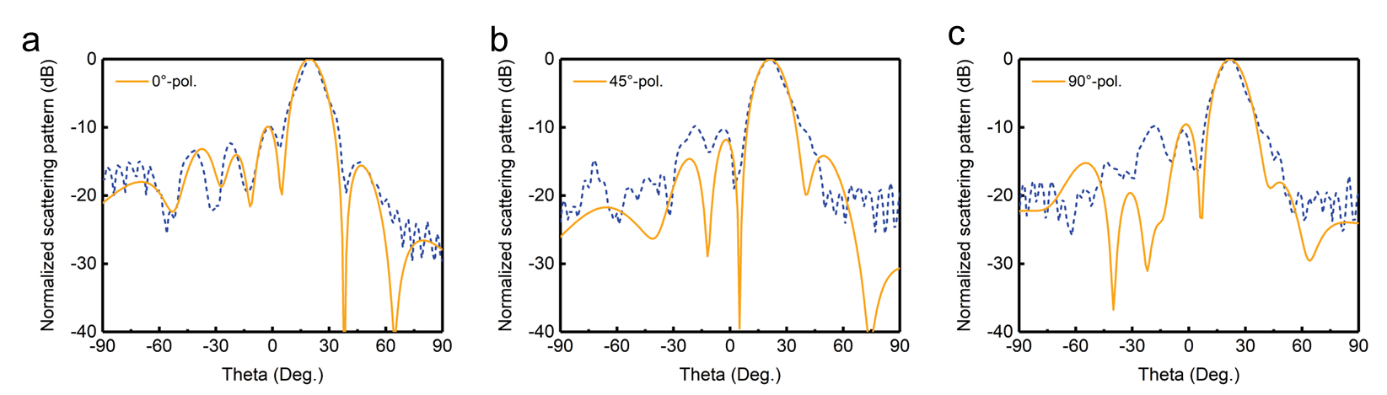


**Fig. S4.** Under *y*-polarized wave excitation, the simulated and measured normalized scattering patterns of (a) 0°-, (b) 45°-, (c) 90°-polarized reflection waves at 3.5 GHz. The polarization is defined in the plane orthogonal to the propagating direction of the beam. The solid lines and the dashed lines indicate the simulated and measured results, respectively.

**Note S5. Manipulation of polarization and beam at the nonlinear frequency.**

2D STPC matrices of dimension 16×4 capable of generating the 0°-, 45°- and 90°-polarized reflection waves are shown in Figure S5a-c, where the modulation frequency is *f*_0_=100 kHz, and the length of the TPC sequence is L= 4. The measured results for the corresponding polarizations at the 0^th^-, +1^st^- and -3^rd^-order harmonics are illustrated in Figures S5d-e, highlighting a drop in scattering directivity for the 0^th^-order harmonic compared to the +1^st^-order. By using Equation (11), the deflection angle of the beam at the +1^st^-, and -3^rd^-order harmonic wave can be calculated as approximately 20.9°, respectively, which is consistent with the measured beam deflection angles of approximately 21° for the different polarized reflection waves.


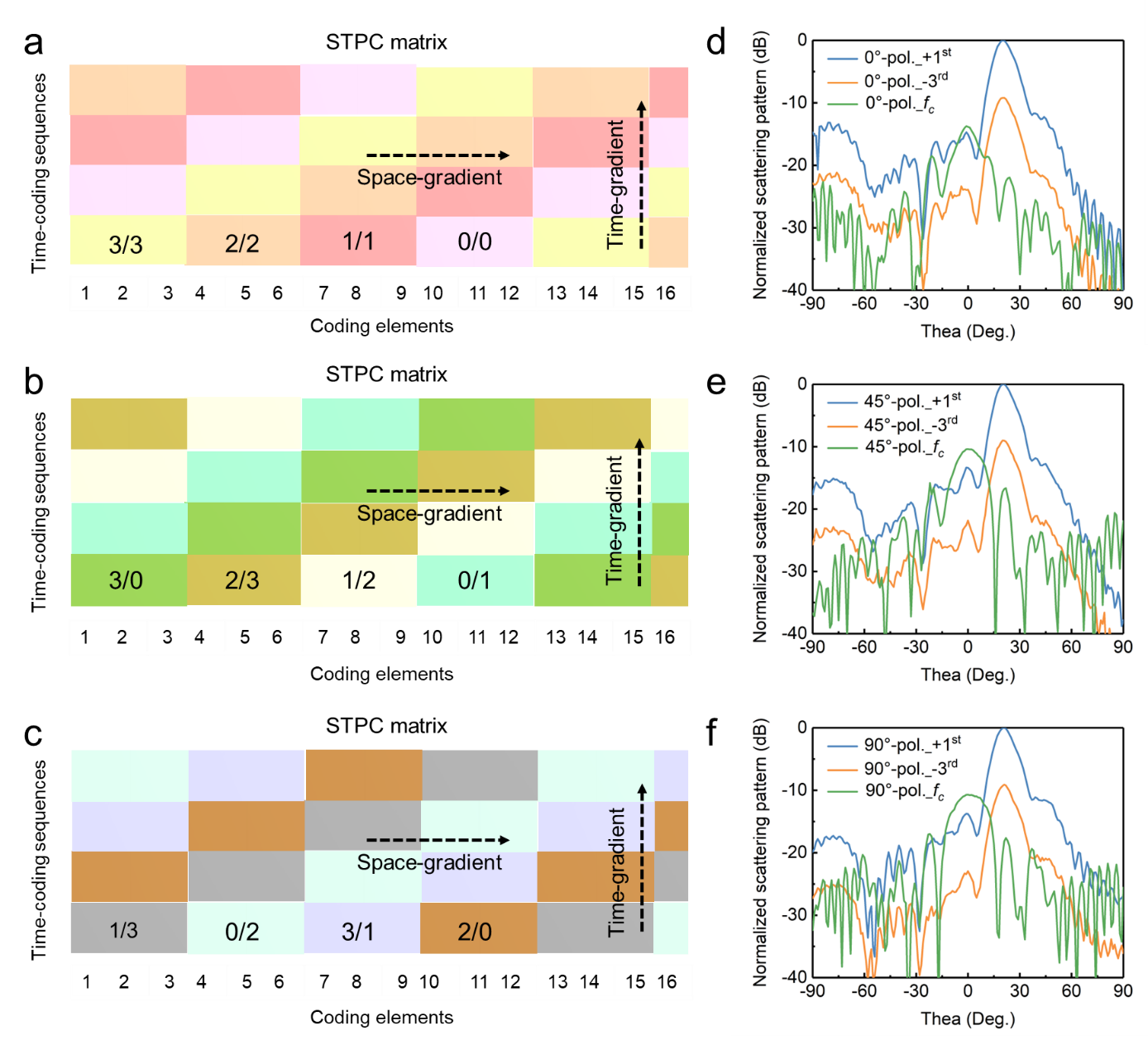


**Fig. S5.** 2D STPC matrices and measured normalized scattering patterns. 2D STPC matrices of dimension 16×4 capable of generating the (a) 0°-, (b) 45°-, and (c) 90°-polarized reflection waves. The measured normalized scattering patterns of (d) 0°-, (e) 45°-, and (f) 90°-polarized reflection waves at the 0^th^-, +1^st^- and -3^rd^-order harmonics. The polarization is defined in the plane orthogonal to the propagating direction of the beam.
